# Lyme Disease Misinterpreted as Child Abuse

**DOI:** 10.1155/2021/6665935

**Published:** 2021-02-01

**Authors:** Tommy Pan, Adam Nasreddine, Myra Trivellas, William L. Hennrikus

**Affiliations:** ^1^Penn State College of Medicine, Hershey, PA 17033, USA; ^2^Yale School of Medicine, New Haven, CT 06510, USA; ^3^UCLA College of Medicine, Los Angeles, CA 90095, USA; ^4^Penn State Hershey Medical Center, Bone and Joint Institute, Hershey, PA 17033, USA

## Abstract

Child abuse is one of the most common causes for child fatality in the United States. Inaccurate reporting of child abuse combined with scarcity of resources for child abuse evaluations can lead to unintended consequences for children and their families. The differential diagnosis of child abuse is varied. To our knowledge, there are no reports in the literature on Lyme disease mimicking child abuse. The current study presents the case of a child from an endemic area for Lyme disease presenting with skin bruising, fracture, and swollen knee. The child was reported for child abuse by the pediatrician and then referred to the orthopaedic surgeon for fracture care.

## 1. Introduction

Child abuse remains a significant problem in the United States. Between the years 2004 and 2011, 5,689,900 children were confirmed cases of maltreatment [1–3]. One in 7 cases of suspected cases of suspected abuse is substantiated [4]. Unidentified abuse can lead both to physical, emotional, and social consequences and is a significant contributor to children fatality [4–6]. It is vital for mandated reporters to accurately recognize and report suspected cases of child abuse.

There are many articles in the medical literature that describe cases of child abuse and highlight the need for reporting. Many clinicians are disconnected from the consequences for families after referral to Child & Youth Protective Services (CYPS) [7]. However, sometimes there is a lack of understanding of the process and outcomes of reported abuse. In many states, social workers from CYPS have 30 days to investigate child abuse cases [8]. CYPS is often understaffed secondary to low staff retention rates and budget limits [8, 9]. Therefore, in some cases, there has been a delay in the completion of investigations as well as a potential decrease of services available to those in need [9, 10]. For example, one author reports of children held in foster care for up to a year pending investigation [4]. Additionally, there remains the ethical dilemma of an innocent person considered guilty until proven otherwise [8, 11].

In the United States, some authors state that child abuse is overreported [9, 11–13]. Public records indicate that about 14% of investigated cases end up confirmed as child abuse [4, 7, 8, 11]. The impetus falls on mandated reporters to accurately identify and report child abuse and also to understand the process and outcomes of reporting. Physicians are unique in their ability to lead the way to identify child abuse correctly. Also, physicians should be aware of evidence-based signs indicative of child abuse as well as possible mimics of child abuse. We present a unique case of a child presenting with Lyme disease mistakenly reported for child abuse.

## 2. Case Report

**Figure 1:**
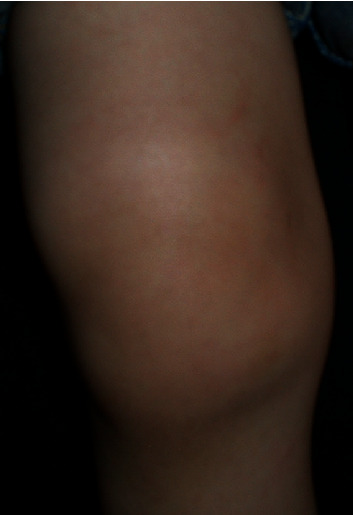
Unilateral knee effusion in a 4-year-old boy presenting to the pediatrician after an unwitnessed fall while playing in the yard.

**Figure 2:**
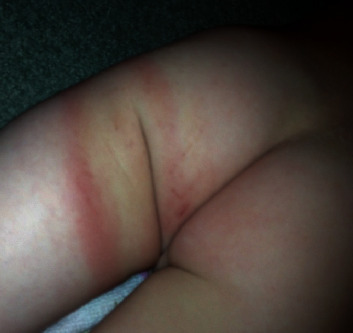
The classic “bull's-eye” rash or erythema migrans of Lyme disease initially mistaken for a thigh bruise of suspected child abuse.

**Figure 3:**
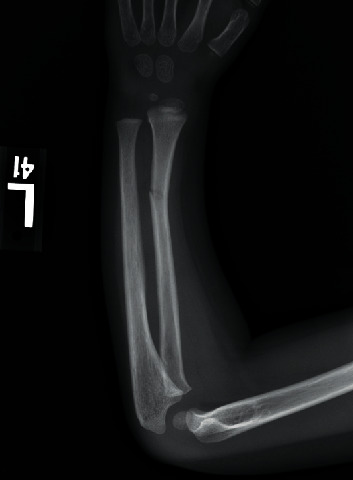
A radiograph demonstrating the left distal radius revealing a minimally angulated greenstick fracture.

**Figure 4:**
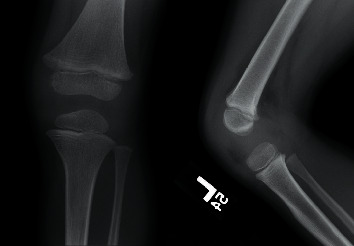
AP and lateral radiographic view of the left knee joint demonstrating joint effusion without presence of fracture.

A 4-year-old boy presented to his pediatrician during late August after an unwitnessed fall playing in his yard. The family lived in a rural wooded area in Pennsylvania. The child was otherwise healthy, no allergies, no medications, no medical illnesses, and one prior surgery—a tonsillectomy. He lives at home with his mother, father, and 3 older siblings. He attends preschool 2 days per week. On presentation to the pediatrician, the patient demonstrated a swollen right knee (Figure 1), a tender left radius without deformity, and what was initially interpreted as a large bruise on the buttock (Figure 2). The skin exam was otherwise negative. The pediatrician obtained radiographs demonstrating a minimally angulated greenstick fracture of the left radius (Figure 3) and a knee joint effusion without fracture (Figure 4). The patient was then referred to the orthopaedic office. Due to the constellation of findings and lack of a witness to the fall, the pediatrician also reported the child to the local County Social Services for Children and Youth due to possible nonaccidental trauma. The child was temporarily placed into foster care.

The orthopaedic surgeon treated the left radius fracture with a long arm cast and noted that despite the swelling of the right knee, the child was afebrile and minimally tender with a 90-degree arc of motion and could bear weight. On careful inspection by the orthopaedic surgeon of the buttock bruising, the skin lesion resembles a “bull's-eye rash”. The mom could not recall if her child had any tick bites; however, the family lived in an area with a large deer population. The child was then sent to the lab for Lyme serology, CBC, and ESR.

Lab results demonstrated a positive Lyme serology via ELISA and Western blot IgM tests, WBC of 9,000 mm^3^ and an ESR of 21 mm/h. Children and Youth ended their investigation of the family, and five days later, they concluded that the child had Lyme disease with an unrelated radius fracture. The child was treated with 30 days of oral amoxicillin per recommendation by the Infectious Disease Society of America [14]. At the orthopaedic office, at follow-up 5 weeks later, the cast was removed and the child's radius fracture was healing uneventfully. The knee effusion and the buttock rash had resolve.

## 3. Discussion

Child abuse remains a cause of child fatality in the United States. Increased public awareness of child abuse along with lack of adequate training for mandated reports can result in overreporting of child abuse cases. Twenty-five percent of child fatality from abuse occurs in families previously reported for child maltreatment [10, 15]. In some cases, children are removed from their families for extended periods of time, only to find that the abuse allegations are not substantiated [4, 16]. Families and individuals under investigation for child abuse often face social isolation which can exacerbate the risk factors for abuse [4, 8, 16, 17]. Lastly, the strategy in investigating child abuse arguably deprive accused individuals of their constitutional right to maintain innocence until proven guilty [8, 11]. Families are subjected to investigation, interrogation, separation, and punishment after a report is made [7]. In the current study, while the cutaneous buttock manifestation is presumed to be indicative of Lyme disease, the radial fracture and knee trauma can potentially be separate events and child abuse may still have taken place. Nevertheless, physicians and other mandated reporters should be aware of the negative consequences of inaccurately reporting child abuse.

Current child protection laws require medical providers among other professionals to report any case of suspected abuse to CYPS. Despite the pivotal role of physicians in accurately identifying and reporting cases with suspected abuse, some physicians receive minimal training to identify and report cases of abuse [4, 18–21]. Levi and Brown reported a significant variability among pediatricians' level of interpreting and understanding of reasonable suspicion for abuse [19]. Ho et al. reported on the low sensitivity of current reporting practices, citing that less than 1 of 7 cases reported by professionals end up as confirmed abuse [4]. Ferrara et al. discussed the impact of a pilot training program for physicians in Italy to help them identify and report cases of child maltreatment. The authors found that training pediatricians on the use of objective signs to supplement their clinical judgment helped to accurately identify more cases of abuse [18]. Orthopaedic surgeons are at the forefront of evaluating and documenting evidence for suspected child abuse because about 28% of confirmed child abuse cases involve one or more fractures [2, 3].

The case in this report is an example of misinterpretation of a constellation of findings including a fracture, swollen knee, and skin bruise. Christian and States and Patel and Butterfield also reported on several medical mimics and skin disorders that can mimic child abuse to the untrained reporter [22, 23].

Several authors have described common objective findings that can be indicative of child abuse [2, 5, 6, 24, 25]. These signs include the following: unexplained injuries such as circumferential burns, human bite marks, bruises on uncommonly injured body surfaces, lacerations or black eyes, fractures in a child who cannot walk or stand, failure to thrive, intra-abdominal trauma, subdural hematomas, retinal hemorrhages, and multiple injuries at different stages of healing. Concurrently, physicians should take into consideration reported mimics of child abuse. In the current case report, we present a child from an endemic area for Lyme disease inaccurately reported for child abuse. Other mimics of child abuse include the following.

Disorders of collagen such as Ehler-Danlos syndrome (EDS), osteogenesis imperfecta (OI), and scurvy can lead to vessel fragility and easy bruising [25–27]. The skin is the largest and most exposed organ [23]. Cutaneous conditions such as congenital dermal melanocytosis (Mongolian spots), capillary hemangiomas, and arteriovenous malformations can masquerade as skin findings of suspected abuse [28, 29]. Heritable hematological disorders such as Bernard-Soulier syndrome (BSS), Glanzmann thrombasthenia (GT), Von Willibrand disease (VWD), hemophilia disorders, and Wiskott-Aldrich syndrome have also been misconstrued for physical abuse [22]. Lichen sclerosus et atrophicus, a chronic inflammatory dermatitis affecting the anogenital region, can mimic findings of sexual abuse in prepubertal girls [23]. The use of over-the-counter moisturizers containing furocoumarin, when exposed to UV radiation, can cause a photooxidative reaction that can present as minor dermatitis to full-thickness scald burns mimicking abuse [30].

In addition, metabolic bone diseases such as rickets and osteopenia can predispose children to fractures mimicking abuse [31]. Children with cholestatic liver disease (CLD) have malabsorption of fat soluble vitamins and increased bone fragility due to poor bone mineralization that can lead to mimics of abuse [31].

Birth trauma such as subdural hemorrhage from vacuum-assisted and cesarean section delivery can also be mistaken for abuse [32]. Furthermore, abusive head trauma (AHT) from “shaken baby syndrome” is another common finding in child abuse [31]. Metabolic disorders such as glutaric aciduria (GA1) or Menkes disease and bleeding disorders such as Factor XIII or vitamin K deficiency can mimic “shaken baby syndrome [33, 34]”.

When reporting suspected child abuse, it is important for physicians to maintain an open mind and consider the differential diagnosis in their evaluations. A prudent medical and family history, detailed physical examination and careful documentation can help differentiate physical abuse from child abuse mimics. Recognizing conditions that masquerade as abuse can improve the diagnostic yield and avoid adverse legal and psychosocial implications on the child, family, and society [23].

## 4. Conclusion

We report a case of Lyme disease with an unrelated distal radius fracture mistakenly reported for child abuse. Physicians should use common sense and employ a systematic approach to evaluate children with suspected abuse. Further research to design a tool to supplement the clinical judgment may be helpful.

## Data Availability

No data were used to support this study.
